# First microsatellite markers for *Paspalum plicatulum* (Poaceae) characterization and cross-amplification in different *Paspalum* species of the Plicatula group

**DOI:** 10.1186/s13104-016-2312-z

**Published:** 2016-12-13

**Authors:** Fernanda A. Oliveira, Fernanda W. Cidade, Alessandra P. Fávero, Bianca B. Z. Vigna, Anete P. Souza

**Affiliations:** 1 Center for Molecular Biology and Genetic Engineering (CBMEG), University of Campinas (UNICAMP), Cidade Universitária Zeferino Vaz, CP 6010, Campinas, SP CEP 13083-970 Brazil; 2 EMBRAPA Southeast Livestock, Brazilian Agricultural Research Corporation, CP 339, São Carlos, SP CEP 13560-970 Brazil; 3 Plant Biology Department, Biology Institute, University of Campinas (UNICAMP), Cidade Universitária Zeferino Vaz, CP 6109, Campinas, SP CEP 13083-970 Brazil

**Keywords:** *Paspalum* botanical group, Germplasm evaluation, Microsatellite, SSR transferability, Forage, Grass

## Abstract

**Background:**

*Paspalum plicatulum* is a perennial rhizomatous grass with natural diploid and polyploid cytotypes. It is a member of Plicatula, which has historically been recognized as a highly complex group containing species of ecological, ornamental and forage importance. The complex nature of the *P. plicatulum* genome makes it a challenging species for genetic research. This study aimed to develop and characterize microsatellite molecular markers in *P. plicatulum* and to evaluate their transferability to other Plicatula group species.

**Findings:**

Microsatellite sequences were identified from three enriched libraries from *P. plicatulum*. Specific primers were designed, and 25 displayed polymorphism when screened across 48 polyploid *Paspalum* spp. genotypes. The number of bands per locus ranged from 2 to 17, with a mean of 8.65. Private bands for each species were identified; the highest number of private bands was observed for *P. plicatulum* in 52% of the loci analyzed. The mean polymorphism information content of all loci was 0.69, and the mean discriminatory power was 0.82. Microsatellite markers were satisfactorily cross-amplified for the eight tested Plicatula-group *Paspalum* species, with *P. atratum* exhibiting the highest transferability rate (89.86%). STRUCTURE and Discriminant Analysis of Principal Components separated accessions into three groups but did not reveal separation of the accessions according to species.

**Conclusions:**

This study describes the first microsatellite markers in *P. plicatulum*, which are polymorphic, efficient for the detection and quantification of genetic variation, and show high transferability into other species of the Plicatula group. This set of markers can be used in future genetic and molecular studies necessary for the proper development of conservation and breeding programs. Private bands within the markers can be used to assist in species identification.

**Electronic supplementary material:**

The online version of this article (doi:10.1186/s13104-016-2312-z) contains supplementary material, which is available to authorized users.

## Background

The genus *Paspalum* L. includes approximately 350 species distributed in tropical and subtropical regions. Most of the species are native to South America [1, 2], including 210 species of Brazilian origin [3]. The basic chromosome number in *Paspalum* is x = 10 [4–6]; this number is the most common, with a few exceptions. Polyploidy is present in nearly 80% of the species [2], with tetraploidy being the most frequent condition (ca. 50%) [7–9], although diploids have also been found in the genus [1, 10]. Generally, species of *Paspalum* consist of sexual-diploid and apomictic-polyploid cytotypes [11].

Among the *Paspalum* species, our efforts are dedicated to the study of *Paspalum plicatulum* Michx., a perennial rhizomatous grass with natural diploid and polyploid cytotypes [12]. This grass originated in Brazil and is widely distributed from the southern United States to southern Argentina and Western India. This species has ecological, ornamental and forage importance [13] and is known as “pasto negro” in Brazil as it is generally used in pastures. *Paspalum plicatulum* is member of the Plicatula group, which represents species that occur throughout the Brazilian territory with wide morphological variation [14].

The Plicatula group is an informal botanic group proposed by Chase [15] to group species related to *P. plicatulum*. The group is easily differentiated by the morphology of the spikelet, which has sterile lemma with transverse wrinkles and a conspicuously convex anthecium and is a shining dark brown. However, the variability of reproductive and vegetative characters makes identification at the specific level difficult. As such, several species and accessions have been included in this group as synonyms for *P. plicatulum* [1]. The lack of correct characterization of the members of this group and the quantification of their variability make it difficult to use them in breeding and conservation programs and in germplasm exchanges. Although some studies have been reported with germplasm banks and taxonomic revisions of this group, many species of the group Plicatula remain unidentified [16, 17].

The complex nature of the *P. plicatulum* genome makes the species a challenging target for genetic research. Thus, our goal in this study was to develop specific microsatellite markers for *P. plicatulum*. Variation of molecular markers specifically developed for the species serves as a powerful tool for the identification of individuals. Furthermore, these markers can potentially provide an excellent tool for the study of the Plicatula group species, aiding in botanical species classification. For this purpose, we isolated and characterized microsatellite markers from *P. plicatulum* and evaluated the transferability of these markers in *Paspalum* species belonging to the Plicatula group.

## Methods

### Plant material and DNA extraction

Three accessions of *P. plicatulum*, BGP 8, BGP 80 and BGP 86 (collector code-V 5852), were chosen randomly for library construction. For characterizing loci, forty-eight *Paspalum* accessions from different species belonging to the Plicatula group were evaluated (Table 1), among which were included two (BGP 8, BGP 80) of the three samples used for the library construction. These samples are from the Germplasm Bank of *Paspalum*, maintained by EMBRAPA Southeast Livestock, São Carlos, SP, Brazil, and were originally collected from the south to the north of Brazil. Twenty-six samples are from *P. plicatulum*, and the other 27 accessions were used for evaluation of transferability and correspond to the following species: 3 from *P. atratum* Swallen; 4 from *P. compressifolium* Swallen; 6 from *P. guenoarum* Arechav., *P. lenticulare* Kunth. and *P. rhodopedum* L.B.Sm. and Wassh. (3); one from *P. lepton* Schult.; and two from *P. rojasii* Hack. Total genomic DNA samples were extracted from lyophilized leaf tissues following the cetyltrimethylammonium bromide (CTAB) method, previously described by [18], and DNA quality and quantity were assessed using 1% agarose gel electrophoresis, with comparison to known quantities of uncut  phage DNA (Invitrogen, Carlsbad, CA, USA).

**Table 1:** Genotypes of *Paspalum* spp. of the Plicatula group used for the characterization and transferability analyses of the new microsatellite markers

AN	Local code (BGP)	Species	Collector code	Latitude	Longitude	Collection site
1	8	*Paspalum plicatulum*	V D Fi [7441]	−27.950000	−50.450000	Capão Alto, Santa Catarina, Brazil
2	67	*Paspalum plicatulum*	V BoPrSe [4258]	−30.116667	−51.966667	Butiá, Rio Grande do Sul, Brazil
3	39	*Paspalum rhodopedum*	V MrFr [9851]	−28.250000	−51.866667	Caseiros, Rio Grande do Sul, Brazil
4	90	*Paspalum plicatulum*	EEA [149]	−30.100000	−51.316667	Guaíba, Rio Grande do Sul, Brazil
5	89	*Paspalum plicatulum*	EEA [148]	−30.100000	−51.316667	Guaíba, Rio Grande do Sul, Brazil
6	88	*Paspalum plicatulum*	EEA [147]	−30.100000	−51.316667	Guaíba, Rio Grande do Sul, Brazil
7	271	*Paspalum lenticulare*	[14535]	−20.679722	−55.296111	Dois Irmãos do Buriti, Mato Grosso do Sul, Brazil
8	220	*Paspalum compressifolium*	V [14196]	−28.833333	−51.566667	Vila Flores, Rio Grande do Sul, Brazil
9	71	*Paspalum plicatulum*	V BoPrSe [4337]	−30.400000	−54.316667	São Gabriel, Rio Grande do Sul, Brazil
10	226	*Paspalum plicatulum*	V, Chies & Palmieri [14206]	−28.283333	−52.450000	Passo Fundo, Rio Grande do Sul, Brazil
11	109	*Paspalum plicatulum*	V BoIrSv [9981]	−32.116667	−52.350000	Rio Grande, Rio Grande do Sul, Brazil
12	172	*Paspalum plicatulum*	V Q FdSv [11893]	−22.933889	−55.635278	Aral Moreira, Mato Grosso do Sul, Brazil
13	83	*Paspalum plicatulum*	V BoPr01 [4741]	−29.000000	−53.666667	Tupanciretã, Rio Grande do Sul, Brazil
14	73	*Paspalum plicatulum*	V BoPrSe [4347]	−30.250000	−54.533333	São Gabriel, Rio Grande do Sul, Brazil
15	232	*Paspalum plicatulum*	V, Chies & Palmieri [14229]	−28.450000	−55.133333	São Luiz Gonzaga, Rio Grande do Sul, Brazil
16	177	*Paspalum plicatulum*	V GoMiSv [11082]	−25.450000	−49.383333	Campo Largo, Paraná, Brazil
17	179	*Paspalum compressifolium*	V GoMiSv [11101]	−25.450000	−49.633333	Campo Largo, Paraná, Brazil
18	180	*Paspalum plicatulum*	V GoMiSv [11102]	−25.450000	−49.633333	Balsa Nova, Paraná, Brazil
19	181	*Paspalum plicatulum*	V GoMiSv [11141]	−25.316667	−49.050000	Campina Grande do Sul, Paraná, Brazil
20	182	*Paspalum plicatulum*	V GoMiSv [11142]	−25.316667	−49.050000	Quatro Barras, Paraná, Brazil
21	261	*Paspalum plicatulum*	[14496]	−18.766667	−51.300000	Itarumã, Goiás, Brazil
22	279	*Paspalum atratum*	V [14554]	−20.483611	−55.806944	Anastácio, Mato Grosso do Sul, Brazil
23	243	*Paspalum plicatulum*	Rc [1333]	−7.350000	−46.600000	Riachão, Paraíba, Brazil
24	265	*Paspalum plicatulum*	V [14503]	−19.566667	−51.233333	Paranaíba, Mato Grosso do Sul, Brazil
25	198	*Paspalum plicatulum*	V GoSv [11450]	−26.516667	−51.916667	Palmas, Tocantins, Brazil
26	197	*Paspalum plicatulum*	VGoSv 11447	−26.516667	−51.916667	Palmas, Tocantins, Brazil
27	301	*Paspalum plicatulum*	VPoRcMmSv 14630	−20.566667	−54.683333	Campo Grande, Mato Grosso do Sul, Brazil
28	164	*Paspalum plicatulum*	VQFdSv 11826	−22.150000	−54.833333	Itaporã, Mato Grosso do Sul, Brazil
29	159	*Paspalum guenoarum*	VQFdSv 12739	−20.633333	−51.100000	Pereira Barreto, São Paulo, Brazil
30	87	*Paspalum plicatulum*	EEA 81	−30.400000	−54.316667	São Gabriel, Rio Grande do Sul, Brazil
31	80	*Paspalum plicatulum*	VBoPrOl 4644	−28.983333	−55.300000	São Borja, Rio Grande do Sul, Brazil
32	137	*Paspalum plicatulum*	VGoMi 10728	−28.833333	−52.433333	Soledade, Rio Grande do Sul, Brazil
33	259	*Paspalum lenticulare*	V 14487	−17.416667	−50.400000	Acreúna, Goiás, Brazil
34	165	*Paspalum lenticulare*	VQFdSv 11827	−22.150000	−54.833333	Dourados, Mato Grosso do Sul, Brazil
35	284	*Paspalum guenoarum*	VRcMmSv 14568	−22.250000	−54.966667	Dourados, Mato Grosso do Sul, Brazil
36	153	*Paspalum compressifolium*	DGoMi 480	−27.383333	−51.133333	Campos Novos, Santa Catarina, Brazil
37	296	*Paspalum rhodopedum*	VRcMmSv 14616	−22.400000	−54.783333	Dourados, Mato Grosso do Sul, Brazil
38	151	*Paspalum rhodopedum*	DGoMi 311	−27.671667	−51.460556	Barracão, Rio Grande do Sul, Brazil
39	249	*Paspalum compressifolium*	VTsOlTf14431	−30.100000	−51.783333	Arroio dos Ratos, Rio Grande do Sul, Brazil
40	407	*Paspalum lepton*	–	–	–	–
41	374	*Paspalum guenoarum*	–	–	–	–
42	212	*Paspalum guenoarum*	–	−30.200000	−56.216667	Quaraí, Rio Grande do Sul, Brazil
43	280	*Paspalum atratum*	VRcMmSv 14557	−20.483611	−55.806944	Anastácio, Mato Grosso do Sul, Brazil
44	35	*Paspalum guenoarum*	VMrFrLw 9813	−28.916667	−55.600000	São Borja, Rio Grande do Sul, Brazil
45	15	*Paspalum atratum*	VPoPrJAr 8687	−20.300000	−56.416667	Miranda, Mato Grosso do Sul, Brazil
46	283	*Paspalum rojasii*	VRcMmSv 14567	−22.250000	−54.966667	Dourados, Mato Grosso do Sul, Brazil
47	264	*Paspalum guenoarum*	V 14502	−19.566667	−51.233333	Paranaíba, Mato Grosso do Sul, Brazil
48	300	*Paspalum rojasii*	VPoRcMmSv 14628	−20.500000	−54.733333	Campo Grande, Mato Grosso do Sul, Brazil

Collectors: Ar = M.R.Araújo, Bo = S.C.Boechat, D = M.Dall’Agnol, Dp = Dario Palmieri, Fd = M.S.França Dantas, Fi = R.G.Fischer, Fr = J.M.O.Freitas, Go = K.E.Gomes, Ir = B.E.Irgang, J = L.Jank, Lw = H.M.Longhi-Wagner, Mi = S.T.S.Miotto, Mm = M.D.Moraes, Mr = C.O.C.Moraes, Ol = M.L.A.A.Oliveira, Po = A.Pott, Pr = A.I.C.Pereira, Q = Camilo Luís Quarín, Rc = Regina Célia de Oliveira, Se = B.A.Severo, Sv = Glocimar Pereira da Silva, Tf = T.F.Ferreira, Ts = T.Souza-Chies, V = José Francisco Montenegro Valls. Abbreviation: EEA = Agronomic Experimental Station of the UFRGS

### Construction of microsatellite-enriched libraries and sequence analysis

For the constructions of the first (Lb-1), second (Lb-2) and third (Lb-3) libraries, we used the accessions BGP 86, BGP 80 and BGP 8, respectively. The libraries were constructed as described in [19]. DNA samples were digested using *Afa*I endonuclease (Invitrogen, Carlsbad, California, USA) and were then ligated to the double-stranded *Afa*I adapters (5′-CTCTTGCTTACGCGTGGACTA-3′) and (5′-TAGTCCACGCGTAAGCAAGAGCACA-3′). An enrichment was performed using hybridization-based capture with (GT)8 and (CT)8 biotinylated probes and streptavidin-coated magnetic beads (Streptavidin Magnesphere Paramagnetic Particles, Promega, Madison, Wisconsin, USA). Selected DNA fragments were amplified by PCR and then cloned into the pGEM-T Easy vector (Promega, Madison, Wisconsin, USA). Competent *Escherichia coli* XL1-Blue cells (Stratagene, Agilent Technologies, Santa Clara, California, USA) were transformed with recombinant plasmids via the electroporation method and were then cultivated on agar medium containing ampicillin (100 mg/ml), X-galactosidase 2% (100 µg/ml) and IPTG (100 mM). Positive clones were randomly selected using white/blue screening and were sequenced on an automated ABI 3500xL Genetic Analyzer (Applied Biosystems, Foster City, California, USA) using T7 and SP6 primers and a BigDye Terminator version 3.1 Cycle Sequencing Kit (Applied Biosystems).

All obtained sequences were analyzed to identify microsatellite-enriched regions with the Simple Sequence Repeat Identification Tool (SSRIT) [20], and oligonucleotides complementary to genomic sequences flanking the microsatellite region were designed using Primer3Plus [21] with the following criteria: preferable primer size between 18 and 22 bp; melting temperature (Tm) between 50 and 60 °C; amplified product length between 100 and 300 bp; and GC content between 40 and 60%. Following these criteria, 56 primer pairs were designed and synthesized for analysis.

### Fragment amplification and statistical analysis

Polymerase chain reactions (PCRs) were performed in a 15-µl final volume containing 30 ng of template DNA, 1× PCR buffer (20 mM Tris HCl [pH 8.4] and 50 mM KCl), 1.5 mM MgCl_2_, 0.2 mM of each dNTP, 10 mg/ml bovine serum albumin (BSA), 0.5 mM of each primer, and 1 U of *Taq* DNA polymerase (Invitrogen, Carlsbad, California, USA). The PCR program used for all loci amplifications was as follows: 2 min 30 s of initial denaturation at 94 °C followed by 35 cycles of denaturation at 94 °C for 1 min, the specific annealing temperature of each primer pair (Table 2), extension at 72 °C for 1 min, and a final extension at 72 °C for 8 min. Amplified products were preliminarily checked on 3% agarose gels prior to genotyping using silver-stained 6% denaturing polyacrylamide gels [22], and product sizes were determined using a 10-bp DNA ladder (Invitrogen, Carlsbad, California, USA). Microsatellites were treated as dominant markers due to the polyploid nature of the genotypes. Accordingly, the data were scored based on the presence (1) or absence (0) of a band for each of the *Paspalum* genotypes. In dominant locus patterns, estimates of allelic frequencies are not possible; therefore, observed heterozygosity was not estimated. As such, polymorphism information content (PIC) was used to evaluate and characterize microsatellite loci using the formula

$$ PIC = 1 - \sum\limits_{i = 1}^{n} {p_{i}^{2} - \sum\limits_{i = 1}^{n} {\sum\limits_{j = i + 1}^{n} {2p_{i}^{2} p_{j}^{2} } } } , $$

where n is the number of bands of the marker among the set of samples used for characterizing the microsatellite polymorphism and pi and pj are the frequencies of bands i and j [23]. Discriminatory power (DP) [24] values were calculated to compare the efficiencies of microsatellite markers in varietal identification.

**Table 2:** Descriptions of SSR markers developed for *Paspalum plicatulum*

Locus name	Source library	GenBank accession number	Reapeat Motif	Ta (C°)^1^	Primer Sequence (5′-3′)	Size Range (bp)	*P. plicatulum*			Plicatula species^a^	
							NA^2^	PIC^3^	DP^4^	NA^2^	PIC^3^
Pp-UNICAMP01	Lb-1	KR611535	(TG)8	60	F: GTGCAACACTATGACACCAG	173–181	4	0.64	0.70	5	0.70
					R: ACAGTGCCCAATTGTTGT						
Pp-UNICAMP02	Lb-1	KR611536	(CGCAC)3	51	F: CTCCACCAACGCCTTAC	187–203	6	0.72	0.81	4	0.45
					R: TAGTCCATACCCTTTCGTTT						
Pp-UNICAMP03	Lb-1	KR611537	(AC)8	60	F: TCTGCTAAGTTACCGCTCAT	127–167	7	0.73	0.87	6	0.65
					R: ATGGATATGGAACTTGATGG						
Pp-UNICAMP04	Lb-1	KR611538	(CA)7	60	F: TTGGATGCACACCAGTATAG	133–151	3	0.41	0.69	7	0.66
					R: CCCTCTTCATTCTCATTCAG						
Pp-UNICAMP05^b^	Lb-1	KR611539	(GT)7	60	F: ATGGATATGGAACTTGATGG	157–173	5	0.61	0.79	6	0.77
					R: CTACGGTCTGCTAAGTCACC						
Pp-UNICAMP06	Lb-1	KR611540	(TC)8	51	F: GGTCCTGGTTGATTGATCT	155–169	7	0.73	0.88	8	0.75
					R: CGGAGTTGAAGATGGACTC						
Pp-UNICAMP07	Lb-1	KR611541	(TCT)4	65	F: AGCCTTGCCTCCAGTC	222–258	6	0.66	0.74	6	0.69
					R: TTTCAGGTTACAGGTTGAGAG						
Pp-UNICAMP08	Lb-1	KR611542	(GT)7	51	F: TGGGTTTGGGATAAGGATAG	144–170	12	0.66	1.00	14	0.69
					R: GGTCCTCAACATGGGTAAC						
Pp-UNICAMP09	Lb-1	KR611543	(AC)7	56	F: GCACAGGTAGAACCATTTCA	228–260	8	0.81	0.85	6	0.78
					R: TATGGAACTTGATGGGATTG						
Pp-UNICAMP10	Lb-1	KR611544	(CA)7	60	F: ATACCTTAGGATCCGCTTCA	230–256	4	0.67	0.83	7	0.72
					R: CACTCTACCGGTCCATGATA						
Pp-UNICAMP11	Lb-1	KR611545	(CA)7	60	F: GGAGAGATGAGACTCCCTTG	232–266	6	0.72	0.86	5	0.69
					R: ACTCTCTACCGGTCCATGAT						
Pp-UNICAMP12	Lb-2	KR611546	(GT)7	65	F: CGCGTGGACTACGATGG	213–277	11	0.79	0.92	14	0.87
					R: AAACGCCCACTCATAATTCG						
Pp-UNICAMP13	Lb-2	KR611547	(CA)3CG(CA)3	55	F: GGAGAGATGAGACTCCCTTGG	116–142	10	0.71	0.74	8	0.63
					R: TCAAGGTGGACCAAACACAC						
Pp-UNICAMP14	Lb-2	KR611548	(ACAT)4	63	F: GATGTTCCGCTCGTTTGATT	223–243	5	0.59	0.75	8	0.67
					R: TGTTGGTCTCAGGCAGCTTAT						
Pp-UNICAMP15	Lb-2	KR611549	(AG)17	55	F: ACAGCTTGGGCCTGACAC	152–166	8	0.74	0.91	7	0.82
					R: GGCTGAACTCTCTTCCATTGTT						
Pp-UNICAMP16	Lb-2	KR611550	(AC)6	55	F: GCACGTGTTCGTCATGAAAT	258–300	4	0.33	0.33	9	0.69
					R: GCTTGGTCCCATGGATTATG						
Pp-UNICAMP17	Lb-2	KR611551	(AG)17	57	F: TGACCGTGCCTAATCTCCTT	147–191	15	0.87	0.99	13	0.86
					R: AAGTTTGCTCTTTCGCGTGT						
Pp-UNICAMP18	Lb-2	KR611552	(GT)8CA(GT)7	65	F: CAGTCAACGACACGGGAAC	119–153	11	0.84	0.98	16	0.90
					R: CCCAACCTAAATCACCTCACC						
Pp-UNICAMP19	Lb-2	KR611553	(ATT)2(CA)8	57	F: CCCTCCCTCCATTTCACA	203–241	13	0.87	0.97	15	0.88
					R: AGCTCGCAGAAGGCAAGA						
Pp-UNICAMP20^b^	Lb-2	KR611554	(GT)7	65	F: AAGAACTGCCAAGGAACT	156–162	4	0.66	0.81	3	0.65
					R: GGAATAAACCTCAATAGGG						
Pp-UNICAMP21	Lb-2	KR611555	(CT)14	55	F: GAGAGCCCAGACACAATGG	145–205	14	0.88	0.99	17	0.90
					R: ATCAACACGCTGCTTCAGTG						
Pp-UNICAMP22	Lb-3	KR611556	(CA)3CG(CA)3	65	F: CGCGGAGAGATGAGACT	133–137	3	0.50	0.64	3	0.35
					R: TCAAGGTGGACCAAACAC						
Pp-UNICAMP23	Lb-3	KR611557	(TA)5A(GT)23	55	F: AGCAGGAGATCATGGAG	230–254	3	0.40	0.57	2	0.35
					R: TCCTACGTGAATGGCTAC						
Pp-UNICAMP24	Lb-3	KR611558	(CA)9	63	F: TCTTGCATCATCCCGTATTG	179–215	10	0.83	0.96	10	0.80
					R: GCTGCCTGGTCCACTCTC						
Pp-UNICAMP25	Lb-3	KR611559	(AC)8	63	F: CGGACCGTCTTTATCTCCAA	218–316	8	0.71	0.92	9	0.78
					R: GCTCCGATCCTCGAAATTCT						

^a^Species from Plicatula group evaluated: *Paspalum plicatulum*, *Paspalum atratum*, *Paspalum compressifolium*, *Paspalum guenoarum*, *Paspalum lenticulare*, *Paspalum lepton, Paspalum rhodopedum and Paspalum rojasii*

^b^ Loci excluded from the statistical analysis due the high index of missing data

^1^Amplification temperature (°C)

^2^Maximum number of alleles observed

^3^Polymorphism information content

^4^Discrimination power

Two approaches were used to evaluate the population structure and grouping of the accessions studied: STRUCTURE and Discriminant Analysis of Principal Components (DAPC).

The model-based Bayesian analysis implemented in the software package STRUCTURE [25] was used to determine the approximate number of genetic clusters (K) within the full data set and to assign individuals to the most appropriate cluster. All simulations were performed using the admixture model, with 500,000 replicates for burn-in and 1000,000 replicates for Markov Chain Monte Carlo (MCMC) processes in ten independent runs. The numbers of clusters (K) tested ranged from 1 to 15. The optimal number of clusters was determined by calculating the ln(K) and ΔK values, as previously described by [26] and as implemented in STRUCTURE HARVESTER [27]. A consensus STRUCTURE plot was obtained from the admixture repeats using the greedy algorithm in CLUMPP [28], and final plots were produced using STRUCTURE PLOT [29].

A DAPC analysis as implemented in the R package *adegenet* was also performed, which uses a nonparametric approach, free from Hardy–Weinberg constraints, as described in [30]. Two approaches were conducted: (1) the first DAPC analysis was performed, providing the information of eight groups according to biological information (eight species); and (2) a priori definition of clusters to study population structure was required; therefore, the number of clusters was assessed using the function *find.clusters*, which runs successive K-means clustering with increasing numbers of clusters (k). We assumed 15 as the maximum number of clusters. The optimal number of clusters was estimated using the Bayesian information criterion (BIC), which reaches a minimum when the best-supported assignment of individuals to the appropriate number of clusters is approached. Both DAPC results are presented as multidimensional scaling plots.

## Results and discussion

For Lb-1, we selected and sequenced 144 positive clones, which yielded 109 contigs containing 33 simple sequence repeat (SSR) sequences. From these sequences, 24 primer pairs were designed and tested. Six were eliminated from the analysis because they did not amplify fragments under the various conditions tested, two had amplification artifacts that made genotyping impossible and five were monomorphic. Thus, 11 polymorphic loci were obtained from Lb-1. In addition to this library, more two genomic libraries were constructed, Lb-2 and Lb-3, with the aim of increasing the number of loci and providing greater robustness in the analysis. A total of 192 positive clones were selected and sequenced, but due to the time required for amplification tests of primer pairs and genotyping, added to the costs involved, we selected only the best sequences of each library to design primer pairs according to the desired parameters. Thirty-two additional primer pairs were designed: 23 from Lb-2 and 9 from Lb-3. From these, four primer pairs were eliminated because they did not amplify fragments, nine had amplification artifacts and five were monomorphic. Thus, more 14 polymorphic loci were obtained (ten from Lb-2 and four from Lb-3).

Twenty-five markers were polymorphic and successfully transferred to the other *Paspalum* species tested. The description of the number of bands per locus and the PIC and DP values for both *P. plicatulum* accessions and other *Paspalum* species are shown in Table 2. Two loci were excluded from statistical analyses because they presented high indices of missing data (Pp-UNICAMP05 and Pp-UNICAMP20). Of the 23 loci analyzed, the number of bands per locus for *P. plicatulum* ranged from 3 to 15, with an average of 7.74. Among the species of the Plicatula group, the number of bands ranged from 2 to 17, with an average of 8.65 bands per locus.

**Fig. 1:**
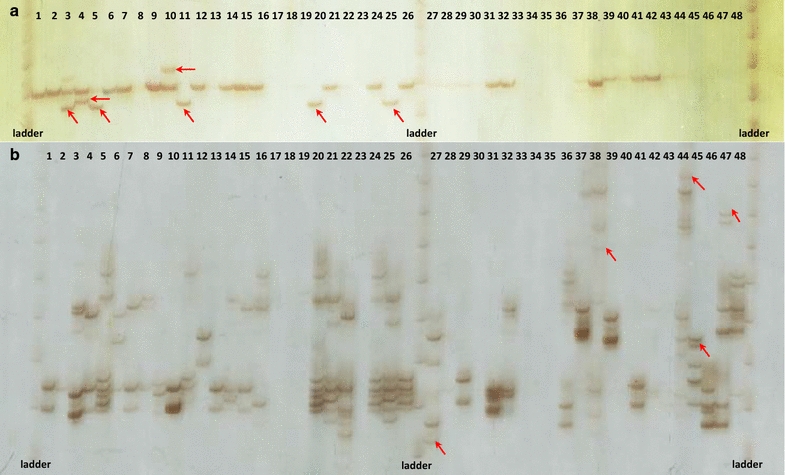
Genotyping of two SSR loci and private bands. Characterization of the **a** Pp-UNICAMP02 and **b** Pp-UNICAMP21 SSR markers resolved in silver-stained 6% denaturing polyacrylamide gel for 48 *Paspalum* genotypes: (*26*) *P. plicatulum*, (*3*) *P. atratum*, (*4*) *P. compressifolium*, (*6*) *P. guenoarum*, (*3*) *P. lenticulare*, (*1*) *P. lepton*, (*3*) *P. rhodopedum* and (*2*) *P. rojasii*, respectively. *Red arrows* highlight the private bands

Private bands were observed for all species, with the exception of *P. rojasii* (Table 3). We use the term “private band” to describe bands that are observed in only one species (Fig. 1). *Paspalum plicatulum* had the highest number of these bands, with more than half of the analyzed loci (52%) showing at least one band unique to this species. *Paspalum atratum* showed the second highest value of private bands, which were observed in 32% of loci. The number of private bands observed, can decrease as more individuals of other species are analyzed. However, this result was interesting since only three individuals of *P. atratum* were analyzed. Private bands in a population are a simple measure of genetic distinctiveness. For the purposes of conservation and management, private bands are crucial for early detection and intervention in populations at early stages of introgression and for prioritizing conservation and recovery programs [31]. Furthermore, a large, reliable, well-chosen set of species-diagnostic markers can be used to help identify species, which is extremely valuable in the management of germplasm banks.

**Table 3:** Numbers of private bands of the 25 SSR markers in *Paspalum plicatulum* and in the seven other *Paspalum* species from the Plicatula group

Locus name	*P. plicatulum*	*P. atratum*	*P. compressifolium*	*P. guenoarum*	*P. lenticulare*	*P. lepton*	*P. rhodopedum*	*P. rojasii*
Pp-UNICAMP01			1					
Pp-UNICAMP02	1							
Pp-UNICAMP03								
Pp-UNICAMP04		1	2					
Pp-UNICAMP05	2	1				2		
Pp-UNICAMP06				1				
Pp-UNICAMP07								
Pp-UNICAMP08		1						
Pp-UNICAMP09	1							
Pp-UNICAMP10		1					1	
Pp-UNICAMP11	1							
Pp-UNICAMP12	3			2				
Pp-UNICAMP13	2							
Pp-UNICAMP14		1			1			
Pp-UNICAMP15	1							
Pp-UNICAMP16		1					1	
Pp-UNICAMP17	5			1	1			
Pp-UNICAMP18				1	3			
Pp-UNICAMP19	1			1				
Pp-UNICAMP20								
Pp-UNICAMP21	2	2					2	
Pp-UNICAMP22								
Pp-UNICAMP23	1							
Pp-UNICAMP24	1	1						
Pp-UNICAMP25	1							
Number of loci with private bands	13	8	2	5	3	1	3	0
% of loci with private bands	52	32	8	20	12	4	12	0

PIC values obtained for the 23 loci analyzed ranged from 0.33 to 0.88, with an average of 0.69 for *P. plicatulum*, and from 0.35 to 0.90, with an average of 0.71 across the species from the Plicatula group. According to the classification proposed by Botstein [32], twenty of our loci were classified as highly informative (PIC ≥ 0.5). DP values ranged from 0.33 to 1.00, with a mean of 0.82 observed for *P. plicatulum*. When the PIC and DP of each locus were analyzed together, five loci presented the highest values in both indexes: Pp-UNICAMP21, Pp-UNICAMP17, Pp-UNICAMP18, Pp-UNICAMP19 and Pp-UNICAMP24, in order of higher informativeness.

Microsatellite markers were satisfactorily cross-amplified for *Paspalum* species within the Plicatula group. *Paspalum atratum* presented the highest detected transferability (89.86%); this species is closely related to *P. plicatulum*. *Paspalum atratum* is highly polymorphic and is related to the “common” biotype of *P. plicatulum* [16]. Killeen [33] relates *P. plicatulum* var. *robustum* Hack. in synonymy to *P. atratum*. The transferability rates of the loci were also high for *P. compressifolium* (85.87%), *P. lenticulare* (82.61%) and *P. guenoarum* (78.26%). The success of transferability between species, as observed for other *Paspalum* species [34, 35] and between correlated grass species [36, 37], allows the reduction of time and costs in the development of new markers. Only the Pp-UNICAMP23 locus could not be amplified in *P. rojasii*. Pp-UNICAMP01 did not amplify in *P. rhodopedum*, *P. rojasii* or *P. lepton*. A total of eight loci did not amplify in *P. lepton*, which may have occurred due to the use of only one accession of this species in the study. The results of the cross-amplification tests are shown in Table 4.

**Table 4:** Cross-amplification of the 25 SSR markers among the other *Paspalum* species evaluated

Locus name	*P. atratum*	*P. compressifolium*	*P. guenoarum*	*P. lenticulare*	*P. lepton*	*P. rhodopedum*	*P. rojasii*
Pp-UNICAMP01	3_3	3_4	2_6	3_3	0_1	0_3	0_2
Pp-UNICAMP02	2_3	4_4	5_6	3_3	0_1	2_3	1_2
Pp-UNICAMP03	2_3	3_4	5_6	3_3	0_1	2_3	1_2
Pp-UNICAMP04	3_3	4_4	4_6	3_3	1_1	2_3	1_2
Pp-UNICAMP05	2_3	3_4	1_6	0_3	0_1	0_3	0_2
Pp-UNICAMP06	3_3	4_4	6_6	3_3	1_1	2_3	2_2
Pp-UNICAMP07	3_3	4_4	5_6	3_3	1_1	3_3	1_2
Pp-UNICAMP08	3_3	4_4	6_6	3_3	1_1	2_3	1_2
Pp-UNICAMP09	2_3	2_4	3_6	2_3	0_1	2_3	1_2
Pp-UNICAMP10	3_3	4_4	5_6	3_3	1_1	3_3	1_2
Pp-UNICAMP11	3_3	4_4	5_6	3_3	1_1	3_3	1_2
Pp-UNICAMP12	2_3	4_4	5_6	3_3	0_1	1_3	1_2
Pp-UNICAMP13	3_3	4_4	5_6	3_3	1_1	3_3	2_2
Pp-UNICAMP14	3_3	4_4	5_6	3_3	1_1	1_3	2_2
Pp-UNICAMP15	2_3	4_4	5_6	2_3	0_1	2_3	2_2
Pp-UNICAMP16	2_3	4_4	4_6	2_3	0_1	2_3	1_2
Pp-UNICAMP17	3_3	4_4	6_6	2_3	1_1	2_3	2_2
Pp-UNICAMP18	2_3	1_4	5_6	3_3	0_1	3_3	1_2
Pp-UNICAMP19	3_3	4_4	4_6	3_3	1_1	3_3	1_2
Pp-UNICAMP20	3_3	4_4	1_6	3_3	1_1	0_3	0_2
Pp-UNICAMP21	3_3	4_4	4_6	2_3	1_1	2_3	1_2
Pp-UNICAMP22	3_3	4_4	3_6	1_3	1_1	1_3	1_2
Pp-UNICAMP23	3_3	1_4	6_6	1_3	1_1	1_3	0_2
Pp-UNICAMP24	3_3	4_4	5_6	2_3	1_1	2_3	2_2
Pp-UNICAMP25	3_3	1_4	5_6	1_3	1_1	1_3	1_2
Total	67	86	110	60	16	45	27
Amplification %	89.33	86.00	73.33	80.00	64.00	60.00	54.00

^a^Number of successfully amplified genotypes_Number of tested genotypes

**Fig. 2:**
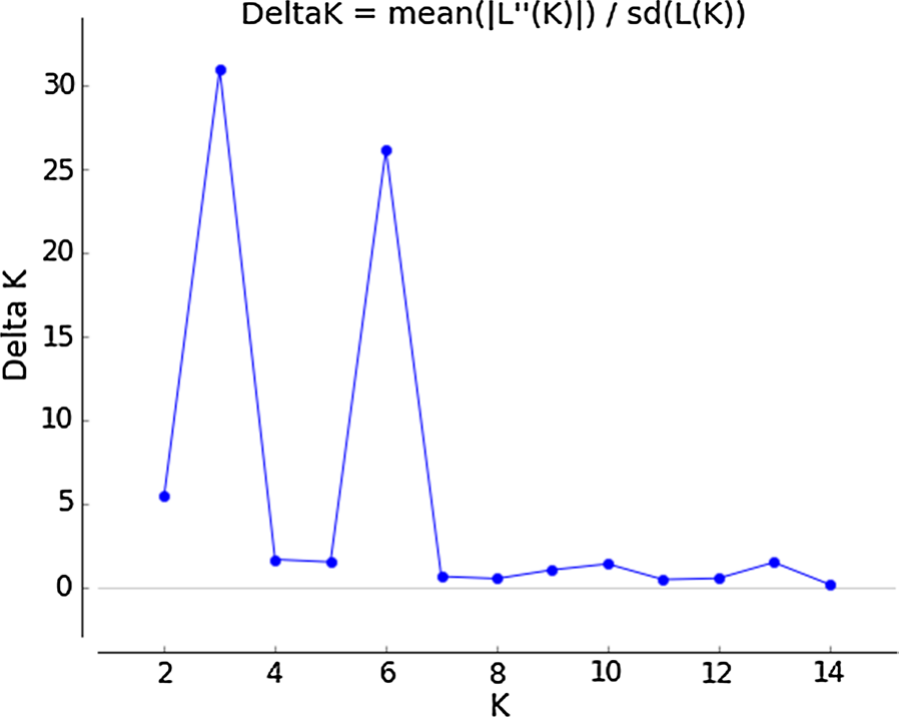
STRUCTURE HARVESTER results. The approximate numbers of genetic clusters (K) within the full data set of 48 individuals based on results from the software package STRUCTURE. The ΔK [25] values are shown for each value of K, from one to fifteen. Results are derived from ten independent simulations for each value of K. The results show the best support for K = 3

**Fig. 3:**
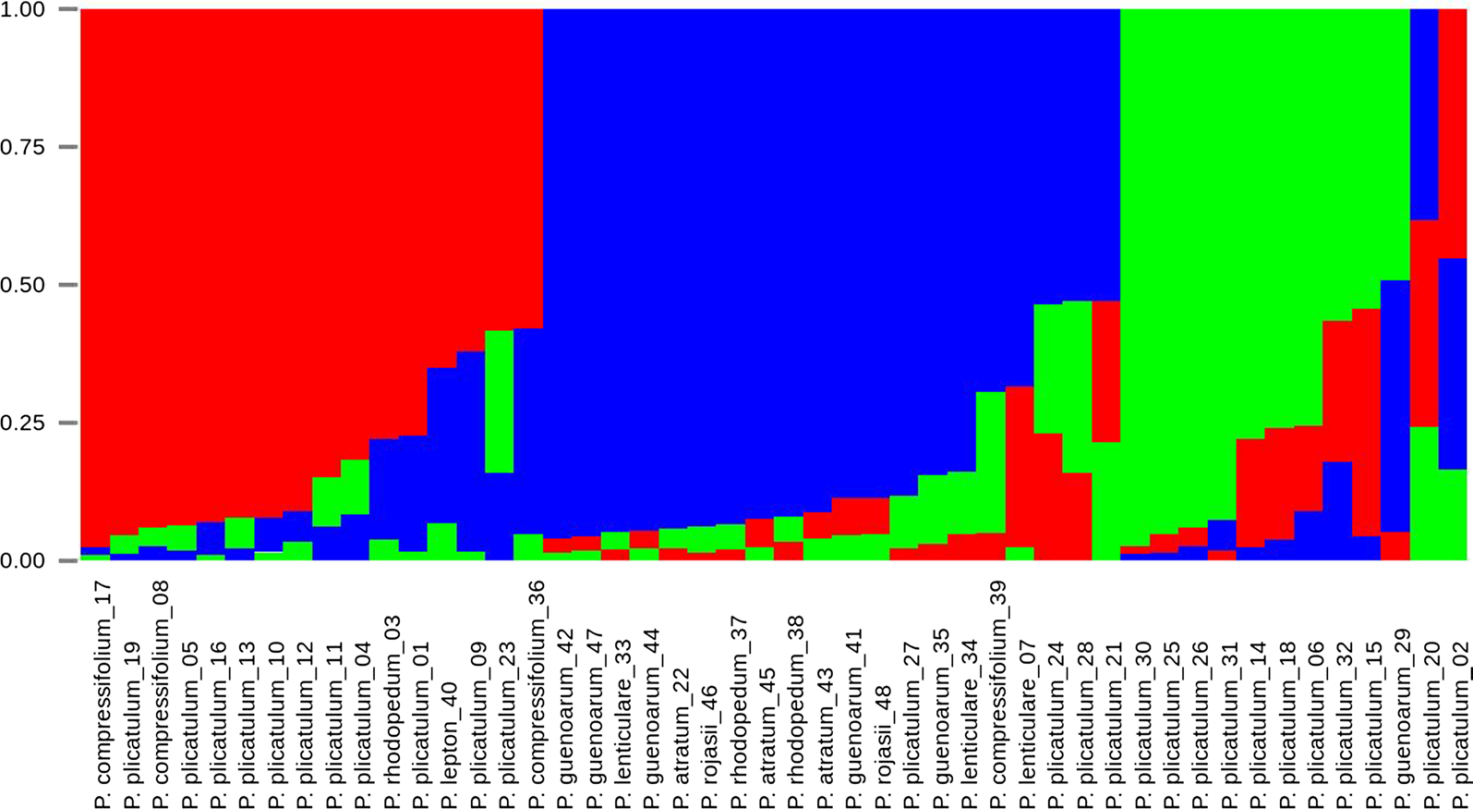
*Bar graph* of the estimated membership coefficient, Q, for each of the 48 individuals. The most likely value of K inferred by STRUCTURE was three. Each genotype is represented by a *vertical bar*, and the colored segments represent the proportion of Q in each of the three clusters (*red*, *blue* and *green*). The data are an average of ten independent runs

The Bayesian analyses of population structure were input into STRUCTURE HARVESTER for computation of mean LnP(K) and ΔK, which peaked at K  = 3, suggesting that three genetic clusters were sufficient to interpret the *Paspalum* germplasm data (Fig. 2); the results are also presented for K = 6, which was the second best K (Additional file 1). From the eight species from Plicatula group analyzed, 16 accessions were assigned to cluster 1 (red), 20 were assigned to cluster 2 (blue) and ten were assigned to cluster 3 (green) (Fig. 3) (Additional file 2). Two accessions of *P. plicatulum* did not sort to defined clusters. There was a tendency of *P. plicatulum* individuals to cluster with each other, but mainly in clusters 1 and 3. The remaining species were mostly classified into a mixed subgroup (cluster 2). However, no clear pattern for the assignment of individuals based on species was observed. The results of this analysis can be extremely useful in breeding programs to guide the choice of accessions to cross. Because the gene pool is shared between accessions of the same cluster, the success rates of crossing them may be higher.

**Fig. 4:**
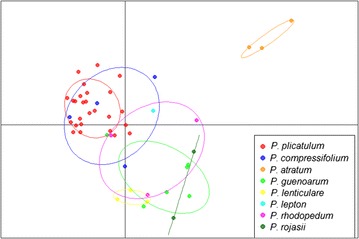
Scatterplots of DAPC using eight species groupings. *Dots* represent individuals, and the species are represented with different colors and inertia ellipses: *P. plicatulum*, *P. compressifolium*, *P. atratum*, *P. guenoarum*, *P. lenticulare*, *P. lepton*, *P. rhodopedum* and *P. rojasii*. DAPC analysis identified two clusters within the data: one large cluster with all individuals from seven *Paspalum* species analyzed and another cluster consisting of *Paspalum atratum* individuals

**Fig. 5:**
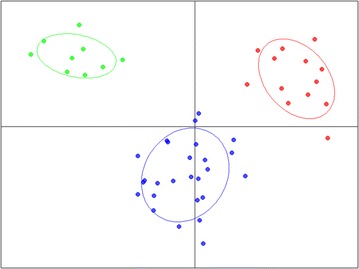
DAPC scatterplots based on the K-means algorithm to determine the proper number of clusters. *Dots* represent individuals, and the clusters are represented with different colors and inertia ellipses. The individuals from different *Paspalum* species were allocated in three clusters: 1 (*red*), 2 (*blue*) and 3 (*green*)

Additionally, two DAPC analyses were performed, and the results are presented as multidimensional scaling plots in Figs. 4 and 5. For both, we retained the first 16 principal components of the PCA, which explained 65% of the variation. In the first approach, we used DAPC to investigate the genetic structure of the sampled individuals, using species information pre-established by taxonomic classification (Fig. 4). The data were divided into two clear clusters: the first cluster consisted only of *P. atratum* individuals, and the second cluster consisted of all individuals from the remaining seven *Paspalum* species. The genetic closeness of these species favors sharing alleles, which complicates delineation between the different taxa of the Plicatula group. This difficulty was also reported by Cidade et al. [17]. However, the separation of *P. atratum* genotypes into a distinct cluster could be related to the efficiency of amplification of the developed loci and the detection of a high number of unique bands. DAPC has been proposed as an alternative to Bayesian clustering [30] as the method does not assume a population genetic model and yields better visualization of the relative distances between groups. In the second approach, we assumed no prior information about groupings of the accessions evaluated. Therefore, we searched for the best-supported number of clusters using the K-means algorithm. Inspection of BIC values (Additional file 3) revealed that two clusters were the most probable to explain the variance in these groups of accessions as the number of clusters should be ≥2, as stated by the software. However, we selected three clusters to illustrate the true genetic clusters and consequently compared the results to the three clusters obtained with STRUCTURE (Fig. 5). The allocation of individuals to clusters from DAPC was similar to those achieved by STRUCTURE, and both analyses showed the same pattern of clustering. Essentially, clusters 1 (red), 2 (blue) and 3 (green) of DAPC reflect the division of gene pools 1 (red), 2 (blue) and 3 (green) detected by STRUCTURE, respectively (Additional file 2).

In both analyses, there was no clear delimitation of taxa within our dataset, compared to that expected according to the number of species studied here. In general, *Paspalum* species of the Plicatula group do not present a clear boundary forming an intricate agamic complex [16]. During species identification, it is common to distinguish among related species using only a few morphological descriptors. In Fig. 3, the unique accession of *P. lepton* was located in cluster 1, where there is a higher concentration of *P. plicatulum* accessions. *Paspalum lepton* is associated with *P. plicatulum* based on general morphological aspects of both plant and spikelet, although the latter species does not possess leptomorph rhizomes. *Paspalum lepton* is also associated with *P. rhodopedum* by the presence of long leptomorph rhizomes, however, the most typical accessions of *P. lepton* are characterized by the presence of small and gray plants [38]. In Fig. 3, the accessions of *P. rhodopedum* appeared to be divided into two clusters. Distinguishing the studied species through taxonomic identification using only morphological characters has proven to be a difficult task. *P. rhodopedum* is strongly related to *P. rojasii*, and it is practically impossible to distinguish the two species without the presence of the base of the plant [16]. Zuloaga et al. [39] proposed the synonymization of these species, but *P. rhodopedum* has leptomorph rhizomes, and *P. rojasii* has pachymorph rhizomes. Zuloaga et al. [39] synonymized *P. guenoarum* and *P. rojasii*. These authors also synonymized *P. rhodopedum* to *P. guenoarum* var. rojasii, even though the first has long and characteristic leptomorph rhizomes [16], as an Israeli chandelier, and *P. guenoarum* does not have rhizomes. Figure 3 shows that accessions of *P. rojasii* grouped with *P. guenoarum* and *P. rhodopedum*. *Paspalum atratum* is morphologically similar to *P. plicatulum* and *P. lenticulare* [38]. One of the morphological characteristics that distinguish *P. atratum* from *P. lenticulare* is that the first has an unbranched stem and the other has a branched colm [16]. The presence of fungi from the genus *Bipolaris* is another morphological characteristic that can be observed in the field for both *P. atratum* and *P. lenticulare*. *P. compressifolium* is a species of glaucous leaves and extremely flat fan-shaped sheaths and is also taxonomically associated with *P. plicatulum*. The accessions of *P. compressifolium* analyzed here showed a different grouping pattern compared to the other species considered in this study. Although the accessions were scattered among themselves and in different clusters, they were grouped with *P. plicatulum* (Fig. 3). However, *P. plicatulum* has wide morphological variability, is poorly understood and there is no consensus in the interpretation of the type specimen. The difficulty in defining this taxon and even the entire Plicatula group was highlighted by Killeen [33].

This result was expected because in a previous study [17], microsatellite markers (SSRs) developed for *P. notatum* and *P. atratum* were used in different *Paspalum* species, which allowed the authors to distinguish many different taxa, except for species belonging to the Plicatula group. Although high genetic variability within its species was observed, there was no clear distinction between different species in this botanical group. Furthermore, it is commonly known that the Plicatula group presents natural variation in morphological types [40], coupled with hybridization throughout its evolution and high genetic variability within its species [17], making it a highly complex group with difficult taxonomic interpretation [17, 33]. However, a more detailed and accurate analysis of the *P. notatum* accessions was undertaken, species from which molecular markers were developed and where species varieties could be separated (var. *saurae* and var. *notatum*) with the aid of private bands. Therefore, we believe that the absence of delimitation between the taxa observed in our study may be related to the low number of genotypes of different species used in the analysis as the initial objective was only to test the transferability of loci.

The private bands identified in our study for seven different species from the Plicatula group present potential applications for species identification of samples and/or new collections, what is mandatory for conservation, cytogenetics, breeding and other uses for *Paspalum*. However, these bands need further validation with larger numbers of genotypes from each species to be brought into practical use.

The microsatellites developed here are the first SSR markers developed for *P. plicatulum* and are highly transferrable to other species of the Plicatula group. These markers showed high polymorphism and were efficient in detecting genetic variations in the different species. These markers can be employed in future investigations of breeding programs, mating systems and kinship studies.

## Additional files

**Additional file 1.** Bar graph of the estimated membership coefficients (Q) by STRUCTURE software for K = 6 for each of the 48 *Paspalum* genotypes evaluated.

**Additional file 2.** Allocation of *Paspalum* individuals based on the groups formed in the analysis of the STRUCTURE and DAPC. Groups 1 (red), 2 (blue) and 3 (green) for both analyses.

**Additional file 3.** Bayesian information criterion (BIC) for different numbers of clusters. The accepted true number of clusters was three.

## Abbreviations

AN: annotation number
BGP: codes of the accessions from EMBRAPA
BIC: Bayesian information criterion
bp: base pairs
BSA: bovine serum albumin
CAPES: coordination of improvement of higher education personnel
CTAB: cetyltrimethyl ammonium bromide
DAPC: discriminant analysis of principal components
DNA: deoxyribonucleic acid
DP: discrimination power
EMBRAPA: Brazilian Agricultural Research Corporation
K: number of clusters
Lb-1: library construction from BGP 86
Lb-2: library construction from BGP 80
Lb-3: library construction from BGP 8
MCMC: Markov Chain Monte Carlo
NA: number of alleles
PCR: polymerase chain reaction
PCA: principal components analysis
PIC: polymorphism information content
Q: association coefficient determined using STRUCTURE analysis
SSR: simple sequence repeat
SSRIT: simple sequence repeat identification tool
Ta (°C): annealing temperature
