# Supplementary material for: First microsatellite markers for Paspalum plicatulum (Poaceae) characterization and cross-amplification in different Paspalum species of the Plicatula group
Source: BMC Res Notes. 2016 Dec 13;9:511. doi: 10.1186/s13104-016-2312-z (PMC5154045; doi:10.1186/s13104-016-2312-z)

Additional file 1. Bar graph of the estimated membership coefficients (Q) by STRUCTURE software for K = 6 for each of the 48 *Paspalum* genotypes evaluated.

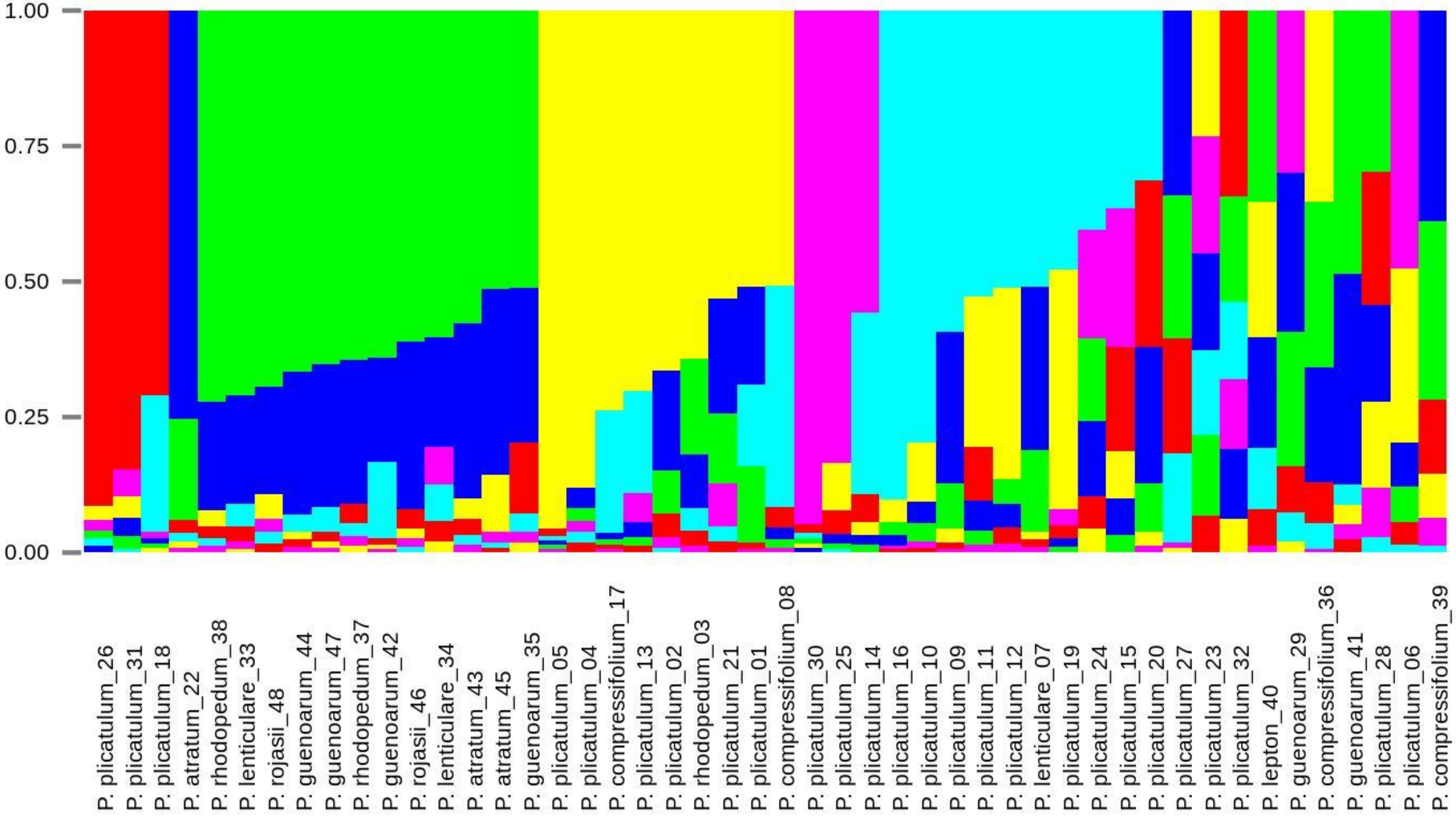

Supplement: Supplementary file 1 — Additional file 1. Bar graph of the estimated membership coefficients (Q) by STRUCTURE software for K = 6 for each of the 48 Paspalum genotypes evaluated. [file 13104_2016_2312_MOESM1_ESM.pdf]
