# Supplementary material for: First microsatellite markers for Paspalum plicatulum (Poaceae) characterization and cross-amplification in different Paspalum species of the Plicatula group
Source: BMC Res Notes. 2016 Dec 13;9:511. doi: 10.1186/s13104-016-2312-z (PMC5154045; doi:10.1186/s13104-016-2312-z)
Supplement: Supplementary file 3 — Additional file 3. Bayesian information criterion (BIC) for different numbers of clusters. The accepted true number of clusters was three. [file 13104_2016_2312_MOESM3_ESM.pdf]

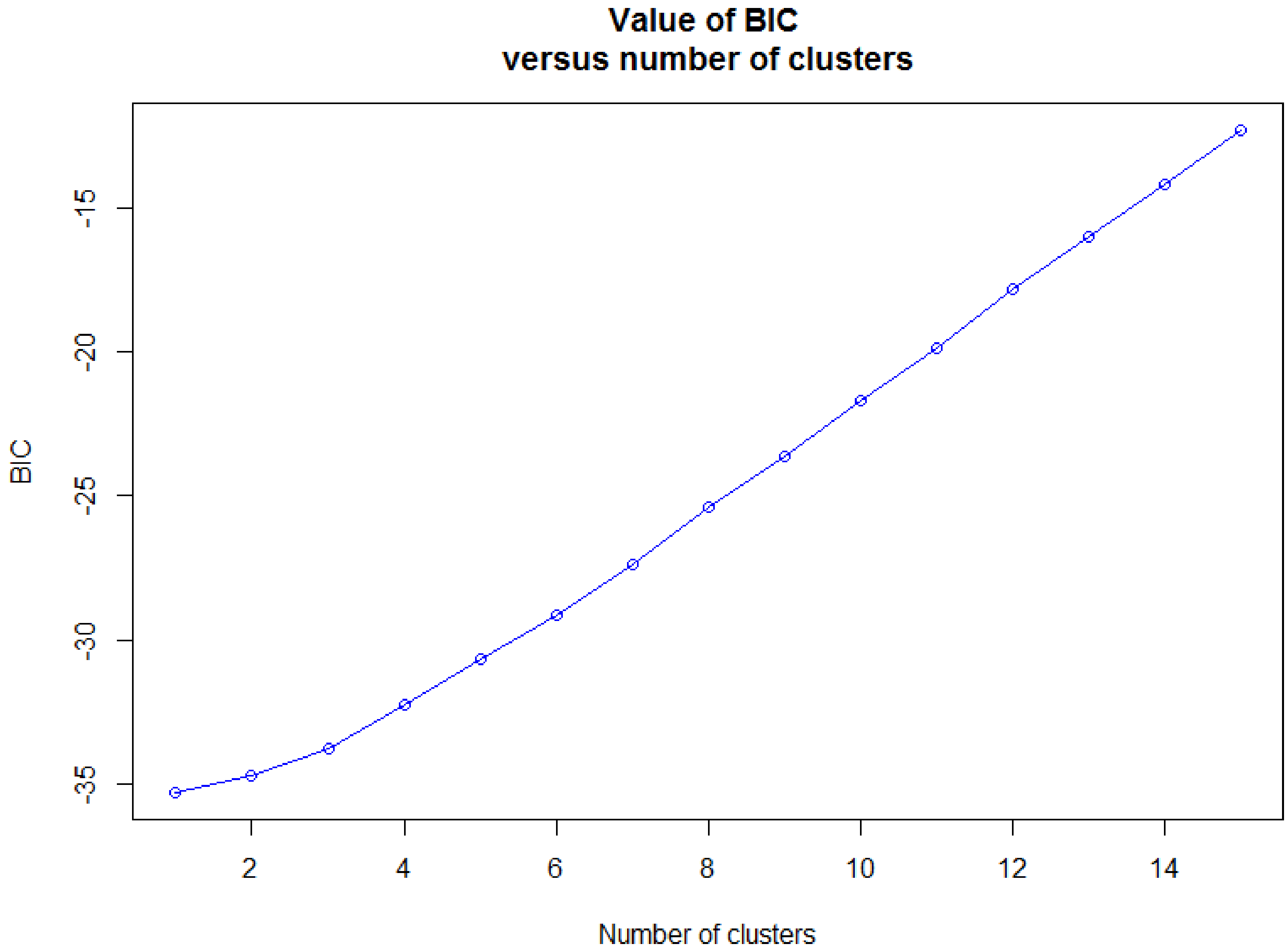

Additional file 3. Bayesian information criterion (BIC) for different numbers of clusters. The accepted true number of clusters was three.
